# ALKBH5 regulates IGF1R expression to promote the Proliferation and Tumorigenicity of Endometrial Cancer

**DOI:** 10.7150/jca.46097

**Published:** 2020-07-25

**Authors:** Xiaowen Pu, Zhuowei Gu, Zhengrong Gu

**Affiliations:** 1 Shanghai First Maternity and Infant Hospital, Tongji University School of Medicine, Shanghai 201204, China.; 2 Department of Obstetrics and Gynecology, Ren Ji Hospital, School of Medicine, Shanghai Jiao Tong University, Shanghai 200127, China.

**Keywords:** ALKBH5, IGF1R, Endometrial cancer, Insulin signaling pathway, m^6^A modification

## Abstract

N6-methyladenosine (m^6^A) messenger RNA methylation play important role in cell proliferation and tumorigenicity of endometrial cancer, but the key mechanism is not fully clear. Here, we found that RNA demethylase ALKBH5 expression was significantly upregulated in endometrial cancer, ALKBH5 was then identified to positively regulate proliferation and invasion of endometrial cancer. Mechanistically, the m^6^A eraser ALKBH5 demethylated target transcripts IGF1R and enhanced IGF1R mRNA stability, consequently promoting IGF1R translation and activating IGF1R signaling pathway. Thus, we demonstrated that ALKBH5 promoted proliferation and invasion of endometrial cancer via erasing IGF1R m^6^A-modifications, which suggests a potential therapeutic target for endometrial cancer.

## Introduction

Endometrial cancer is the most prevalent malignancy of the female genital tract in many countries. Women have a 2-3% lifetime risk of developing this malignancy 1. The majority of endometrial cancer patients diagnosed at early stage and cured by surgery resulted in 5-year survival rate of 95% but women diagnosed with advanced or recurrent disease have a poor prognosis with 5-year survival rate of 17%, which seriously affect the health of women 2. However, the available treatment options after initial therapy at the time of disease progression or recurrence are still limited 3. Therefore, better understanding of the molecular mechanism of endometrial cancer and developing new therapies based on molecular signatures are needed to improve patient's survival rate, but the key mechanisms mediating tumorigenicity of endometrial cells are not fully clear.

*N^6^*-methyladenosine (m^6^A) is the most prevalent messenger RNA modification in eukaryotes. The biological effects of this reversible modification are mostly through methylase, demethylase and association recognize proteins 4-7. m^6^A modification is reported to participated in regulating RNA structure 8, translation 9 and degradation 10. m^6^A-dependent mRNA regulation affects diverse biological processes in mammals 11.* Alkbh5* knockout mice have shown compromised spermatogenesis 7. m^6^A carried mRNA regulates the differentiation of stem cells by affecting mRNA stability and regulating transcriptome switching during embryonic development 12-14. Consistent with these roles, m^6^A mRNA methylation also affects cancer initiation and progression in a variety of cancers 15-17. Recently reported that reduced m^6^A mRNA methylation as an oncogenic mechanism in endometrial cancer and identify m^6^A methylation as a regulator of AKT signaling 17. However, how the key target genes of ALKBH5 affect endometrial cell activity and the underlying pathways mediate these changes are still far from elucidated.

“Insulin like growth factor (IGF) axis” plays a key role in regulating cell growth and survival and affecting virtually every organ in the body 18-20. Clinical and experimental data identified the insulin-like growth factors (IGF1, IGF2) as important players in endometrial tumors in particular 21. Both IGF1 and IGF2 ligands activate the cell-surface tyrosine kinase receptor IGF1 receptor (IGF1R), which coupled to several intracellular secondary messenger pathways, including the Ras/Raf/MAPK and PI3K/AKT signaling cascades 22. The IGF1R is vital for cell survival, as illustrated by the lethal phenotype of the IGF1R KO mice. Elevated IGF1R expression was observed in 91.3% of endometrial cancers, consistent with the key role of the IGF in cancer progression, IGF1R and IGF2 levels were much higher in advanced stage malignant tissue compared to early stages or endometrial hyperplasia, which indicated that over-expression of the IGF1R and IGF2 genes are associated with poor outcome in endometrial cancer 23. Moreover, insulin/IGF1R signaling can induce the expression of membrane-type matrix-metalloproteinase, which is the key regulator of cell invasion, migration and tissue remodeling 24, 25. It is reported that P53 and BRCA1 can regulated the IGF1R expression 26, 27. However, how to accurately regulate the expression of IGF1R, especially via the RNA epigenetic way, subsequently affecting the downstream signaling of IGF1R, still needs to be illustrated.

Herein, we found that RNA demethylase ALKBH5 expression was significantly upregulated in endometrial cancer clinical samples. ALKBH5 knockdown decreased the expression of key molecular IGF1R and inhibited the activation of insulin-like growth factor signaling pathway and inhibited proliferation and invasion of endometrial cancer *in vitro*. Together, our results may add insights to the m^6^A demethylase ALKBH5 as an important regulator of the insulin-like signaling pathway in promoting proliferation and invasion of endometrial cancer, providing potential targets to prevent the development of endometrial cancer.

## Methods and Materials

### Patient sample collection and protein/RNA extraction

All samples were obtained with informed consent under a protocol approved by the ethics committee of Shanghai First Maternity and Infant Hospital, China. The study is compliant with all relevant ethical regulations regarding research involving human participants. All Endometrial cancer samples were diagnosed and assessed in accordance with the International Federation of Gynecology Oncology (FIGO) criteria (2009). All the Endometrial cancer samples must have been diagnosed as endometrial adenocarcinoma and underwent initial surgery. All patients had no history of malignancies. Clinical and pathological data of all patients were retrieved from their records at our department. Patient's characteristics are listed in Table 1. Fresh endometrial tumors and adjacent normal endometrium were separately dissected at the time of surgery and immediately transferred to RNAlater (Thermo Fisher, AM7021). Tissues were homogenized in TRIzol reagent. Total RNA and protein were extracted following the manufacturer's instructions.

### Reagents

Antibodies used for experiment were as follows: β-Actin (sc-8432,), and HRP-coupled secondary antibodies (sc-2749) were from Santa Cruz Biotechnology (Santa Cruz, CA, USA). ALKBH5 (ab174124) antibody was from Abcam (Cambridge, UK). IGF1R (#9750) antibodies were from CST company. Antibody specific to m^6^A (202003) was from Synaptic Systems.

### Cell culture and transfection

HEC-1-A cell lines were obtained from American Type Culture Collection (ATCC, Manassas, VA, USA) and cultured as grown in McCoy's 5A medium (Gibco, 16600) supplemented with 10% fetal bovine serum (FBS) (Gibco), and 1% penicillin-streptomycin (Gibco). The RL95-2 cells were purchased from ATCC and grown in DMEM: F-12 medium supplemented with 10% FBS, 0.005 mg/mL insulin, and 1% penicillin-streptomycin. The T-HESCs were purchased from ATCC and grown in DMEM: F-12 medium supplemented with 10% FBS, 1% ITS-premix, 1 mM pyruvate, 0.5 µg/mL puromycin and 1% penicillin-streptomycin. Cells were transfected with siRNAs (final concentration: 20 nM) and plasmids with Lipofectamine™ 2000 (ThermoFisher, American) according to the manufacturer's instructions. All siRNAs were obtained from GenePharma shown below: IGF1R sense #1: GCGGAGAGAUGUCAUGCAATT, antisense #1: UUGCAUGACAUCUCUCCGCTT, sense #2: GCUUCACCGUUUACUACAATT, antisense #2: UUGUAGUAAACGGUGAAGCTT; Negative control, Sense: UUCUCCGAACGUGUCACGUTT, Antisense: ACGUGACACGUUCGGAGAATT.

To establish stably transfected HEC-1-A cells, G418 was added (1000 μg/mL) 48 h after transfection and maintained at 800 μg/mL for 3 weeks for positive selection. For stably transfected cells, ALKBH5 and mutant H204A expression were confirmed by Western blot. Lentiviruses harboring human ALKBH5 shRNAs were purchased from OBIO technology (Shanghai, China). HEC-1-A cells were infected with the lentivirus (MOI: 10:1). After HEC-1-A cells were transfected for 72 h, 5 μg/mL puromycin was added for 72 h and maintained at 1 μg/mL for 3 weeks for positive selection of stably knockdown ALKBH5. The sequences encoding the short-hairpin RNA are shown below: Human Alkbh5, #1: TCGTGTCCGTGTCCTTCTT, #2: GACTGTGCTCAGTGGATAT; Human Mettl3, CGTCAGTATCTTGGGCAAGTT; Negative control, TTCTCCGAACGTGTCACGT.

### RNA fractionation, RNA extraction and RT-qPCR

HEC-1-A cells were transfected with indicated shRNA, siRNAs or plasmids. 48 h post-transfection, total RNA was extracted by TRIZOL reagent (Invitrogen). RNA was reversed-transcribed using the Reverse Transcription System from Toyobo (Osaka, Japan). The reverse transcription products from different samples were amplified by real-time PCR and analyzed as described previously 28. The primer sequences for Q-PCR analysis are listed below: Alkbh5 Forward: CGGCGAAGGCTACACTTACG, Reverse: CCACCAGCTTTTGGATCACCA; IGF1R Forward: AGTGCTGTATGCCTCTGTGAACC, Reverse: ATAGACCATCCCAAACGACCC; Mmp9 Forward: TCCACCCTTGTGCTCTTCCC, Reverse: CTGCCACCCGAGTGTAACCAT; Col1a1 Forward: GTGCTTATTGGTTCTCCGTTAGT, Reverse: CACAAGCCAGAAATCCTCCAT; Hprt Forward: CCTGGCGTCGTGATTAGTGAT, Reverse: AGACGTTCAGTCCTGTCCATAA; Actb Forward: CATGTACGTTGCTATCCAGGC, Reverse: CTCCTTAATGTCACGCACGAT; Gapdh Forward: GCCAAGGTCATCCATGACAACTTTGG, Reverse: GCCTGCTTCACCACCTTCTTGATGTC.

### Transwell migration and invasion assay

For invasion assays, cell-culture inserts (0.8 μm, Falcon no. 353097) were pre-coated with Matrigel (40 μg/insert, Corning) in serum-free medium for 30 mins at 37 °C. For migration assays, inserts were not pre-coated. 40,000 cells per insert were seeded in the upper chamber of the insert and cultured in 300 μL RPMI-1640 medium supplemented with 2% FBS. Complete RPMI -1640 medium supplemented with 10% FBS was used in the lower chamber. Following 16-24 h of migration or invasion, fluorescent stain (calcein-AM) was added to each lower chamber and incubated for 30 mins. Images were collected with a Nikon Eclipse Ti2 with NIS Elements imaging software (version 5.02) and images analyzed with ImageJ (version 1.51i).

### Colony formation assay

500 cells were seeded per well in six-well culture dishes. After 7 to 10 days, the culture medium was removed and the cells were washed twice with PBS, fixed with 4% paraformaldehyde for 20 min, stained with 0.1% crystal violet (in 25% methanol) for 20 min, washed with water and dried. Colonies were counted manually.

### Cell proliferation assay

5000 cells were seeded per well in a 96-well plate. The cell proliferation was assessed by assaying the cells at various time points using the Cell counting kit-8 (Sigma-Aldrich, 96992) following the manufacturer's protocols. For each cell line tested, the signal was normalized to the value observed about 24 h after seeding.

### m^6^A-qRT-PCR

The procedure was adapted from the previous report 29. For m^6^A-qRT-PCR, total RNAs were firstly subjected to mRNA purification by Poly(A) selection (FastTrack MAG Micro mRNA isolation kit, invitrogen), but not randomly fragmented to facilitate RT with oligo dT and PCR amplification. Then it was performed as described in m^6^A-seq assay but without generating cDNA library. For comparing m^6^A abundance changes, relative enrichment was first normalized with inputs, and then analyzed by comparing the data from m^6^A-immunoprecipitated sample. All samples were analyzed in triplicate qPCR.

### Measurement of RNA lifetime

HEC-1-A cells were seeded in 24 well plates at 60% confluency. After 24 h, actinomycin D (A4262, Sigma) was added to 5 mg/mL 6 h, 4 h, 2 h and 0 h before collection. The total RNA was purified using an RNeasy kit with an additional DNase-I digestion step on the column. RNA quantities were determined by qRT-PCR. The HPRT1 gene was used as a reference gene when carrying out qPCR.

### Molecular cloning of related genes

Related genes were obtained from human endometrial cell by RT-PCR and subsequently cloned into pcDNA vectors. Each construct was confirmed by sequencing. The corresponding primers used in this study are listed below:

- Alkbh5 Forward: CCGGAATTCATGGCGGCCGCCAGCGGCTAC,
- Reverse: GCCGGTACCTCAGTGCCGCCGCATCTTCACCTTT;
- Alkbh5 (H204A) Forward: GGCTGCATCGTGTCCGCCGTGGACCCCATCCA,
- Reverse: GCCGGGCTGGTAGTCGTTGATGACGGCGC;
- IGF1R Forward: CCGGAATTCATGAAGTCTGGCTCCGGAGGAGG,
- Reverse: CGCGGATCCTCAGCAGGTCGAAGACTGGGGC.

### Immunoblot

Cells were lysed using Cell Lysis Buffer (Cell Signaling Technology) supplemented with cocktail protease inhibitor (Calbiochem). Protein concentrations of the extracts were measured using BCA assays (Pierce, USA) and equalized with the extraction reagent. Equivalent amounts of extract were loaded to SDS-PAGE, transferred onto PVDF membranes, and then blotted as described previously 30.

### Statistical analysis

All experiments were independently repeated at least three times. Comparisons between two groups were performed using Student's *t*-test. Data were analyzed with GraphPad Prism Software. Statistical values achieving *p*<0.05 were considered to be statistically significant.

## Results

### ALKBH5 expressed highly in the endometrial cancer

**Figure 1:**
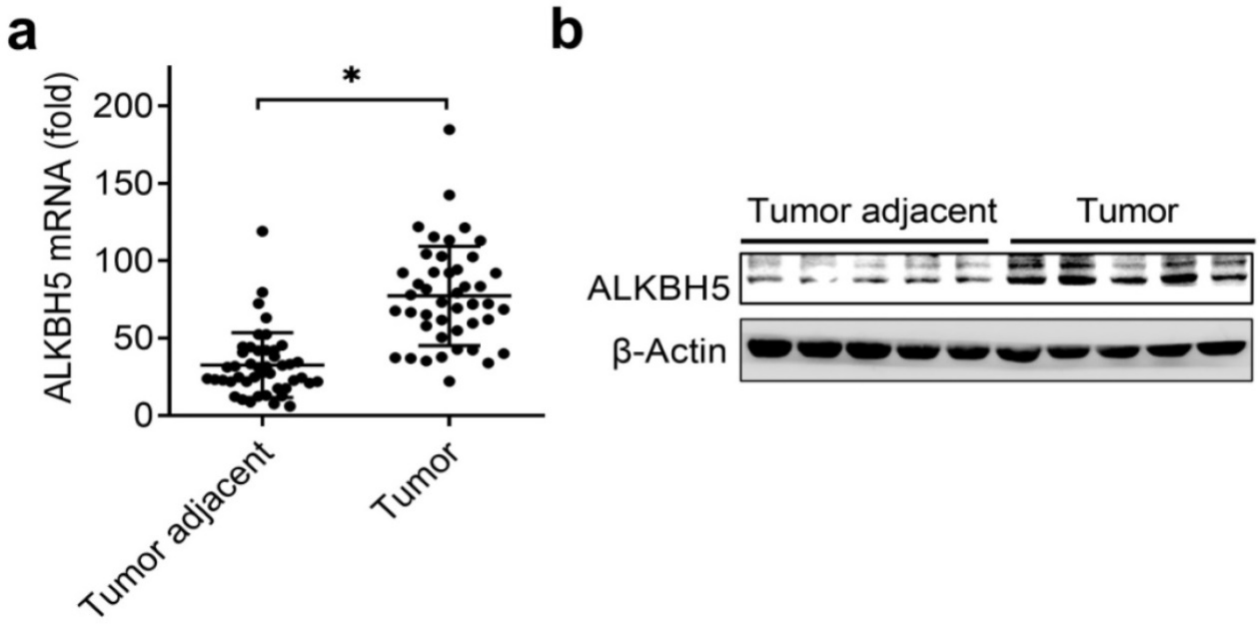
** Expression of ALKBH5 significantly upregulated in endometrial cancer.** (**a**) qRT-PCR analysis of ALKBH5 mRNA in endometrial cancer (Tumor) (n=45) and endometrial cancer adjacent (Tumor adjacent) (n=45) clinical samples. The gene expression was normalized to that of the β-Actin internal control in each sample. (**b**) Immunoblot analysis of ALKBH5 in endometrial cancer (Tumor) (n=5) and endometrial cancer adjacent (Tumor adjacent) (n=5) clinical samples. *p<0.05 (Student's t-test). Data are representative of three independent experiments (mean and s.d. of technical triplicates (**a**)).

The previously paper reported that reduced m^6^A mRNA methylation as an oncogenic mechanism in endometrial cancer and identify m^6^A methylation as a regulator of AKT signaling 17, we predicted that the m^6^A modification associated enzymes maybe the key regulators of the endometrial cancer. Intriguingly, we identified that the mRNA levels of RNA m^6^A demethylase ALKBH5 were significantly upregulated in endometrial cancer compared to adjacent normal endometrium (Figure 1A and Table 1). We further confirmed that the protein levels of ALKBH5 were significantly upregulated in endometrial cancer (Figure 1B). Taken together, these results indicated that upregulation of ALKBH5 may play an important role in the tumorigenesis and progression of endometrial cancer, and led us to further examine how ALKBH5 could regulate endometrial cell activity.

### ALKBH5 promoted the activity of endometrial cell

**Figure 2:**
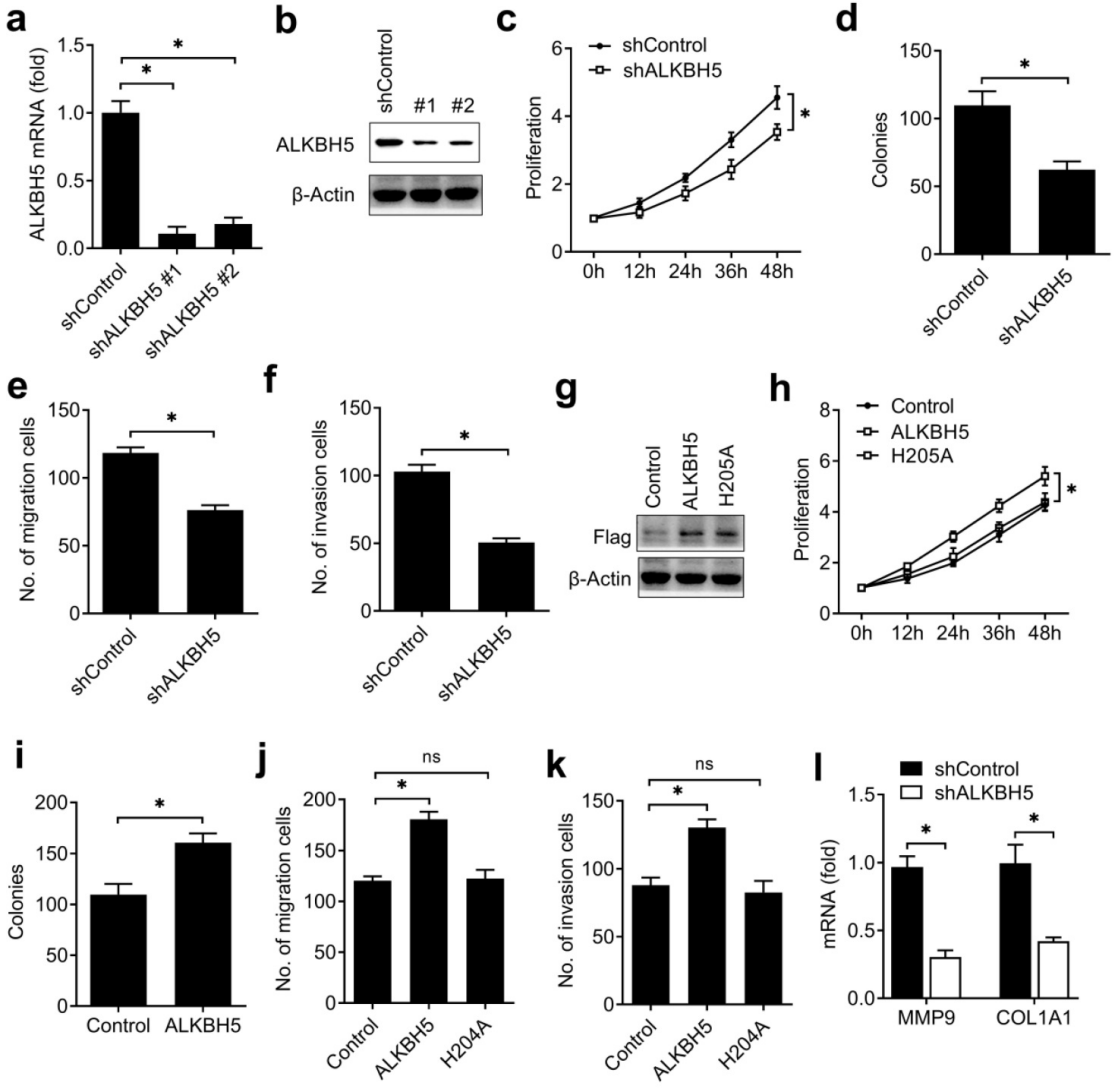
** ALKBH5 promoted the activity of endometrial cell.** (**a,b**) qRT-PCR and immunoblot analysis of ALKBH5 in endometrial cell HEC-1-A transfected with lentiviral shControl, ALKBH5 shRNA #1 or #2 as indicated for 72 h. (**c,h**) Cell proliferation measured by CCK8 assay of HEC-1-A cells stably expressing shALKBH5 (**c**) and ALKBH5 or H204A (**h**) as indicated. The value of OD _450_ was normalized to the value about 24 h after cell seeding. (**d,i**) Colony formation analysis of HEC-1-A cells stably expressing shALKBH5 (**d**) or ALKBH5 (**i**) as indicated. (**e,f**) Migration (**e**) and invasion (**f**) analysis of HEC-1-A cells stably expressing control shRNA (shControl) or shRNA targeting ALKBH5 (shALKBH5) by Transwell assay. The numbers of migration and invasion cell were counted by ImageJ software. (**g**) Immunoblot analysis of HEC-1-A cells stably expressing ALKBH5 or H204A as indicated. (**j,k**) Migration (**j**) and invasion (**k**) analysis of HEC-1-A cells transfected with ALKBH5 or H204A by Transwell assay. The numbers of migration and invasion cell were counted by ImageJ software. (**l**) qRT-PCR analysis of MMP9 and COL1A1 mRNA in HEC-1-A stably expressing shControl and shALKBH5 as indicated, and stimulated with IGF for 8 h. *p<0.05 (Student's t-test). Data are representative of three independent experiments (mean and s.d. of technical triplicates (**a,c-f,h-l**)).

To investigate the biological functions of ALKBH5, we examined the effects of ALKBH5 knockdown on endometrial cell activity. Firstly, we confirmed that ALKBH5 was stably knocked down by using two different lentiviral shRNAs sequences (Figure 2A, B). We found that ALKBH5 knockdown significantly inhibited the proliferation, migration and invasion of endometrial cell (Figure 2C-F). In parallel, overexpression of wild-type ALKBH5 significantly enhanced the proliferation, migration and invasion of endometrial cell, while overexpression of ALKBH5 demethylation-inactive H204A mutant had no noticeable effect on the activity of endometrial cell (Figure 2G-K), suggesting that ALKBH5 could promote the activity of endometrial cell depending on its demethylation activity.

To further determine the downstream genes and signal pathway of ALKBH5 in regulating the activity of endometrial cell, we confirmed that knockdown of ALKBH5 inhibited collagen type I alpha 1 chain (COL1A1) and the matrix metallopeptidase 9 (MMP9) expressions in HEC-1-A (Figure 2L). These results indicated that ALKBH5 promoted the activity of endometrial cell probably via enhancing the MMP9 and COL1A1 expression.

**Figure 3:**
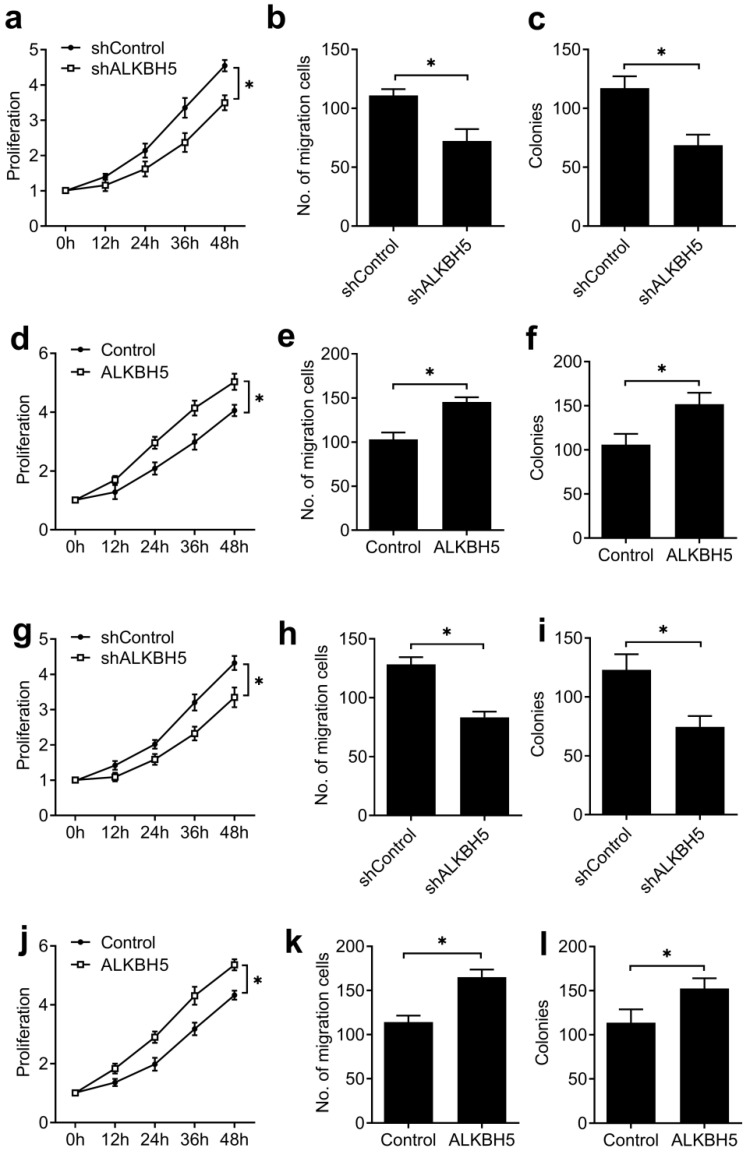
** ALKBH5 promoted the activity of endometrial cell RL95-2 and normal non-transformed endometrial cells (T-HESCs).** (**a,d,g,j**) Cell proliferation measured by CCK8 assay of endometrial cell RL95-2 (**a**,**d**) and T-HESCs (**g**,**j**) transfected with lentiviral ALKBH5 shRNA or ALKBH5 plasmid as indicated. (**b,e,h,k**) Migration analysis of endometrial cell RL95-2 (**b**,**e**) and T-HESCs (**h**,**k**) transfected with lentiviral ALKBH5 shRNA or ALKBH5 plasmid as indicated by Transwell assay. The numbers of migration cell were counted by ImageJ software. (**e,f,i,l**) Colony formation analysis of endometrial cell RL95-2 (**e**,**f**) and T-HESCs (**i**,**l**) transfected with lentiviral ALKBH5 shRNA or ALKBH5 plasmid as indicated. *p<0.05 (Student's t-test). Data are representative of three independent experiments (mean and s.d. of technical triplicates (**a-l**)).

To further confirm that our results extend beyond the HEC-1-A endometrial cancer cell line, we tested the effects of knockdown and overexpression of ALKBH5 in a second endometrial cancer cell line (RL95-2), as well as human endometrial stromal cells (T-HESCs), a normal non-transformed cell line, and found similar effects on the activity of endometrial cell (Figure 3), these results furtherly indicated that ALKBH5 could promote the activity of endometrial cell.

### IGF1R transcripts with demethylation in endometrial cancer

**Figure 4:**
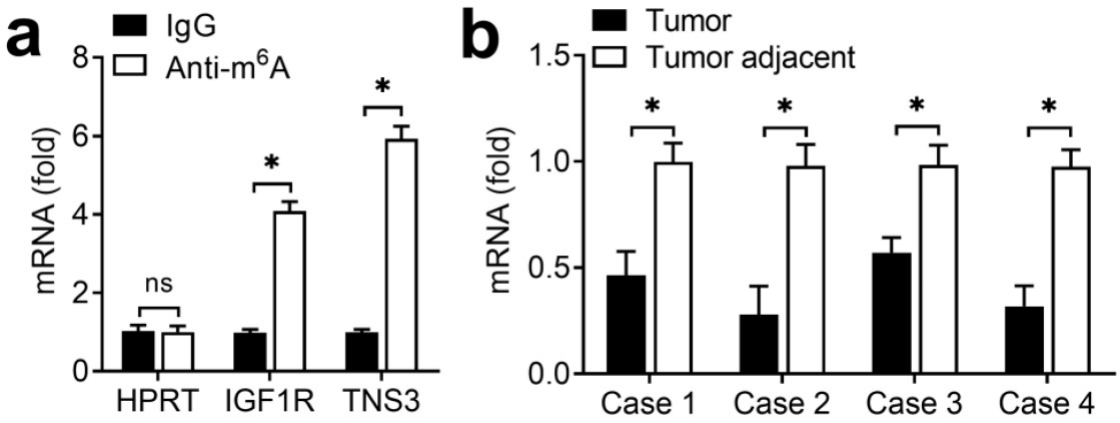
** m^6^A methylation of IGF1R mRNA downregulated in endometrial cancer.** (**a**) Enrichment fold of the indicated mRNA transcripts in m^6^A IP versus mRNA input control. (**b**) Enrichment fold of the IGF1R mRNA transcripts in m^6^A IP versus mRNA input control in endometrial cancer tissue as indicated. Each transcript was quantified by qRT-PCR. ns: not significant; *p<0.05 (Student's t-test). Data are representative of three independent experiments (mean and s.d. of technical triplicates (**a,b**)).

Due to that the regulation of ALKBH5 on endometrial cell activity depended on its demethylation activity, we intended to identify the transcripts with demethylation in endometrial cancer. Previous m^6^A-seq results showing decreased m^6^A methylation were enriched in KEGG terms related to focal adhesion (IGF) signaling pathway 17. Because the IGF signaling pathway played an important role in the development of endometrial, we hypothesized that ALKBH5 might promote the activity of endometrial cell through activation of the IGF pathway. Intriguingly, we found that IGF1R of IGF signaling pathway had m^6^A methylation modifications according to m^6^A-seq data 17. We performed immunoprecipitation of the HEC-1-A mRNA with m^6^A antibody followed by RT-qPCR, and found that, in addition to negative control HPRT, IGF1R and TNS3 (positive control) mRNAs were enriched in the m^6^A antibody-bound fraction, confirming the presence of m^6^A sites on the IGF1R transcripts (Figure 4A). IGF1R showed decreased enrichment in the m^6^A antibody-bound fraction of the endometrial cancer when compared with adjacent normal endometrium (Figure 4B), thus indicating that m^6^A sites on IGF1R mRNAs disappeared in the endometrial cancer. These results revealed that ALKBH5 regulated the activity of endometrial cell probably via demethylating IGF1R transcripts in IGF signaling pathway.

### ALKBH5 catalyzed m^6^A demethylation of IGF1R mRNA and promoted IGF1R expression

**Figure 5:**
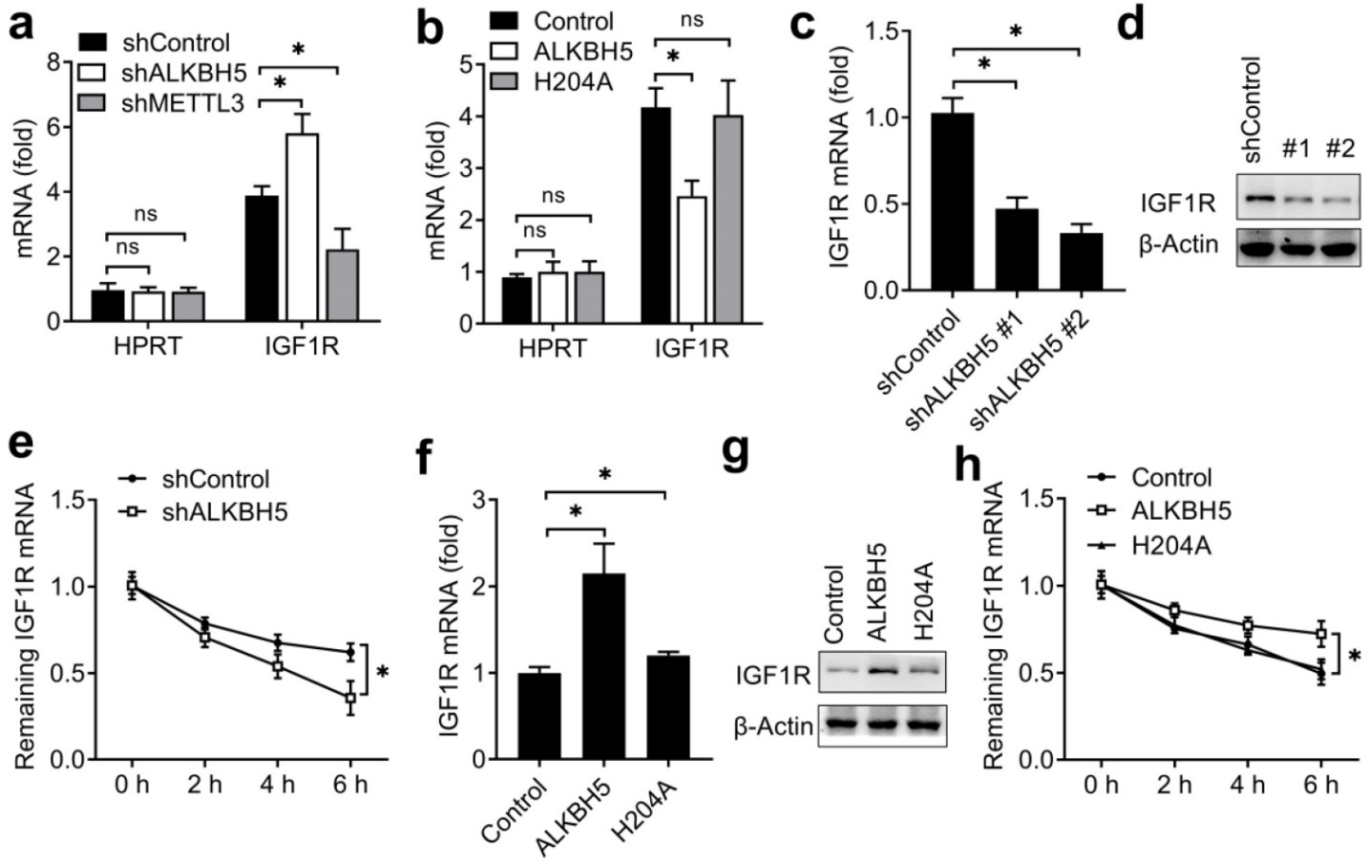
** ALKBH5 catalyzed m^6^A demethylation of IGF1R mRNA and promoted IGF1R expression.** (**a**,**b**) Enrichment fold of the indicated mRNA transcripts in m^6^A IP versus mRNA input control in HEC-1-A after transfected with the indicated shRNA for 72 h (**a**) or plasmids (**b**) for 48 h. Each transcript was quantified by qRT-PCR. (**c**,**d,f,g**) qRT-PCR and immunoblot analysis of the IGF1R mRNA and protein in HTR-8/SVneo transfected with shALKBH5 for 72 h(**c,d**) or plasmids as indicated for 48 h (**f,g**). (**e**,**h**) RNA lifetime for IGF1R in HEC-1-A cells transfected with shALKBH5 (**e**) or plasmids (**h**) was determined by monitoring transcript abundance after transcription inhibition (TI). ns: not significant; *p<0.05 (Student's t-test). Data are representative of three independent experiments (mean and s.d. of technical triplicates (**a**-**c**,**e**,**f**,**h**)).

As ALKBH5 is an m^6^A eraser of RNA methylation, we intend to investigate whether ALKBH5 targets the m^6^A modification of IGF1R mRNA. We performed immunoprecipitation of the HEC-1-A mRNA with m^6^A antibody, and found that, IGF1R showed increased enrichment in the m^6^A antibody-bound fraction when ALKBH5 was knocked down, but decreased enrichment when the m^6^A 'writer' METTL3 was knocked down (Figure 5A), thus indicating that m^6^A sites on IGF1R mRNAs were the direct substrates subject to ALKBH5-catalyzed demethylation. Furthermore, the enrichment of IGF1R mRNAs to m^6^A antibody decreased in HEC-1-A after overexpression of wild-type ALKBH5, but not altered after overexpression of an ALKBH5 mutant lacking demethylation activity (H204A) (Figure 5B). These observations further support that ALKBH5 can erase m^6^A modification of its substrates IGF1R.

We next explored that whether the expression of IGF1R were affected after erasing m^6^A modification. We found that knockdown of *ALKBH5* decreased the mRNA and protein levels of the *IGF1R* transcripts and IGF1R transcripts showed increased RNA decay rates upon knockdown of *ALKBH5* (Figure 5C-E). Overexpression of ALKBH5, but not its H204A mutant, increased the mRNA and protein levels of the *IGF1R* transcripts and IGF1R transcripts showed decreased RNA decay rates upon ALKBH5 overexpression (Figure 5F-H). These results indicated that ALKBH5 promoted the expression of IGF1R via inhibiting the decay of IGF1R transcripts.

### ALKBH5 regulated the activity of endometrial cell via targeting IGF1R

**Figure 6:**
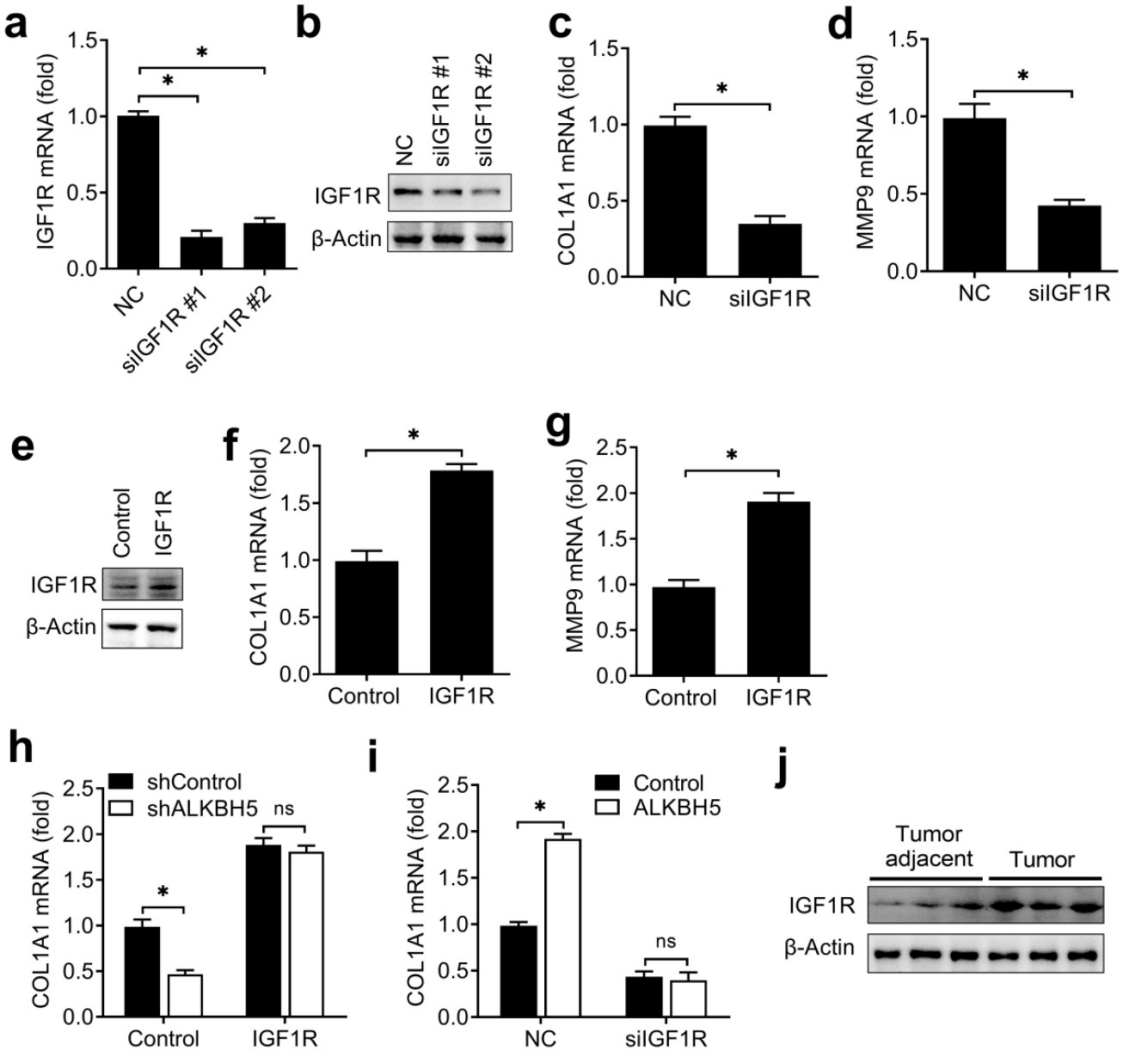
** ALKBH5 regulated the activity of endometrial cell via targeting IGF1R.** (**a**,**b**) qRT-PCR and immunoblot analysis of IGF1R in HEC-1-A transfected with negative control (NC), IGF1R siRNA #1 or #2 as indicated for 48 h. (**c**,**d,f,g**) qRT-PCR analysis of COL1A1 or MMP9 mRNA in HEC-1-A transfected with siRNA (**c,d**) or plasmids (**f,g**) as indicated, and stimulated with IGF for 6 h. (**e**) Immunoblot analysis of IGF1R in HEC-1-A transfected with the IGF1R plasmids for 48 h. (**h**) qRT-PCR analysis of COL1A1 mRNA in stably expressing shALKBH5 HEC-1-A transfected with plasmids as indicated, and stimulated with IGF (50 ng/mL) for 6 h. (**i**) qRT-PCR analysis of COL1A1 mRNA in stably expressing ALKBH5 HEC-1-A transfected with siRNAs as indicated, and stimulated with IGF (50 ng/mL) for 6 h. (**i**) Immunoblot analysis of IGF1R in endometrial cancer (Tumor) (n=3) and endometrial cancer adjacent (Tumor adjacent) (n=3) clinical samples. ns: not significant; *p<0.05 (Student's t-test). Data are representative of three independent experiments (mean and s.d. of technical triplicates (**a**,**c**,**d**,**f**-**i**)).

We then investigated the potential roles of IGF1R in regulating the activity of endometrial cell. Firstly, we confirmed that IGF1R was efficiently knocked down by two different siRNAs (Figure 6A,B). We found that knockdown of IGF1R significantly inhibited the COL1A1 and MMP9 expression of endometrial cell (Figure 6C,D) when compared with negative control, and overexpression of IGF1R significantly promote the COL1A1 and MMP9 expression of endometrial cell (Figure 6E-G), which was a phenocopy of our results obtained from ALKBH5. We next asked whether ALKBH5 regulated the activity of endometrial cell mainly through IGF signaling. We found that ALKBH5 overexpression-mediated promotion of COL1A1 production was rescued by IGF1R knockdown (Figure 6H), suggesting that IGF1R knockdown counteracted the effect of ALKBH5 overexpression. Accordingly, overexpression of IGF1R reversed the ALKBH5 knockdown-mediated inhibition of COL1A1 production (Figure 6I), thus determining that the role of ALKBH5 in promoting endometrial cell COL1A1 production was dependent on IGF1R.

To investigate whether the expression of IGF1R protein in endometrial cancer adjacent and endometrial cancer were consistent with the ALKBH5, we found that IGF1R were indeed upregulated in endometrial cancer (Figure 6J). Taken together, we found that ALKBH5 regulated the activity of endometrial cell mainly via targeting IGF1R.

On the basis of our findings, we propose the following working model to explain how ALKBH5 promotes the activity of endometrial cell. ALKBH5 expression significantly upregulates in endometrial cancer, and ALKBH5 demethylate the m^6^A modifications of IGF1R transcripts, and promotes IGF1R expression leading to the activation of the IGF signaling pathway, which consequently induces COL1A1 and MMP9 expression, subsequently increases the invasion and migration of endometrial cells.

## Discussion

We find that the RNA demethylase ALKBH5 expression upregulated in endometrial cancer. Downregulation of ALKBH5 can inhibit endometrial cell proliferation and invasion *in vitro*. Mechanically, ALKBH5 promotes the expression of IGF1R via demethylating m^6^A modification and increase the expression of COL1A1 and MMP9, which can promote the invasion of endometrial cell. Together, our results indicate that the m^6^A demethylase ALKBH5 maybe a potential target to prevent the tumorigenicity of endometrial cancer.

It is reported that about 70% of endometrial tumours exhibit reduced m^6^A methylation when compared with matched normal endometrium, which were probably caused by either mutation of METTL14 or reduced expression of the METTL3 methyltransferase 17. We also found that ALKBH5 expression was upregulated in endometrial cancer, this maybe also the important reasons for endometrial cancer with reduced m^6^A methylation, this is consistent with previous observations that ALKBH5 was significantly upregulated in many cancer tissues 31. Using the TCGA databases to analyze the expression and survival of ALKBH5 in uterine corpus endometrial carcinoma (UCEC), we found that the expression of ALKBH5 was downregulated in primary tumor compared to normal tissue and the effect of ALKBH5 expression level on UCEC patient survival is not significantly (data not shown). These results have some differences with our findings - we speculate that the reason for the difference is that our analysis is relative to the matched adjacent tumor tissues, while TCGA analysis is relative to the unmatched normal tissues.

Either METTL14 mutation or METTL3 downregulation could reduce m^6^A mRNA levels and enhance proliferation of endometrial cancer cells in vitro, m^6^A-seq of endometrial cancer patient tumors and cell lines revealed that reduced m^6^A mRNA methylation could promote cell proliferation by altering the expression of key enzymes that affect the AKT signaling pathway 17. We also found that overexpression of ALKBH5 could enhanced the proliferation, migration and invasion of endometrial cell, and this activity depending on its demethylation activity. ALKBH5 promoted the activity of endometrial cell via upregulation of COL1A1 and MMP9 expression, which finally promoted the progression of endometrial cancer.

The dynamically m^6^A methylated mRNA could have effects on cellular physiology, especially if the key transcripts are affected 32. We know that ALKBH5 is an m^6^A demethylase; here we found that the key transcripts IGF1R in IGF signaling pathway were demethylated upon ALKBH5 overexpression, and accordingly the decay of IGF1R mRNAs were inhibited after demethylation. These results indicated that the m^6^A methylation as an important factor promoting cancer progression, reveal that reduced m^6^A mRNA methylation on the key transcript is most likely an oncogenic mechanism underlying mostly endometrial cancers and identify m^6^A methylation as a regulator of the cell growth.

Changes in IGF1 expression and signaling play key roles in the regulation of normal uterine physiology 22. Regulation of IGF1R expression and action by tumor suppressors (p53, BRCA1) involved in the etiology of gynecological cancer has a major impact on cell's activity 33. Regulation of IGF1R expression is an important mediator of these changes to endometrial cell invasion mediated by ALKBH5. Increased IGF1R expression is probably one of the main mediators of increased activity in cells with ALKBH5 overexpression, as overexpression of IGF1R is sufficient to rescue the inhibition of the COL1A1 expression mediated by ALKBH5 knockdown. These findings may be applicable beyond endometrial cell invasion to other cancers driven by ALKBH5 overexpression. However, we cannot rule out the involvement of other signaling pathways that could be altered directly or indirectly by change of ALKBH5, this need to be further investigated.

## Figures and Tables

**Table 1:** Tissue characteristics of patient with endometrial cancer

Characteristics	Numbers	Percent (%)
**Age (years)**		
31-40	1	2.2
41-50	8	17.8
51-60	21	46.7
61-70	13	28.9
71-80	2	4.4
**FIGO 2009 Staging**		
I	34	75.5
II	7	15.6
III	3	6.7
IV	1	2.2
**Grade**		
G1	26	57.8
G2	14	31.1
G3	5	11.1
